# Basal Cell Carcinoma Cleft: The Missing Piece of the Puzzle

**DOI:** 10.7759/cureus.71244

**Published:** 2024-10-11

**Authors:** Elena Niculet, Diana S Radaschin, Manuela Arbune, Carmen Bobeica, Mihaela Craescu, Cristian Onisor, Gabriela Gurau, Camelia Busila, Claudiu I Vasile, Alin L Tatu

**Affiliations:** 1 Department of Pathology, "Sfantul Apostol Andrei" Emergency Clinical Hospital of Galati, Romania, Galati, ROU; 2 Department of Morphological and Functional Sciences, Faculty of Medicine and Pharmacy, “Dunărea de Jos” University, Galati, ROU; 3 Multidisciplinary Integrated Center of Dermatological Interface Research (MIC-DIR), “Dunărea de Jos” University, Galati, ROU; 4 Department of Dermatology, Saint Parascheva Infectious Disease Clinical Hospital, Galati, ROU; 5 Biomedical Doctoral School, “Dunărea de Jos” University, Galati, ROU; 6 Department of Clinical Medicine, Faculty of Medicine and Pharmacy, “Dunărea de Jos” University, Galati, ROU; 7 Department of Clinical Medicine, Faculty of Medicine and Pharmacy, “Dunarea de Jos” University, Galați, ROU; 8 Department of Dermatology, “Sf. Cuvioasa Parascheva” Clinical Hospital of Infectious Diseases, Galati, ROU

**Keywords:** basal cell carcinoma, cleft, clefting, histopathology, tumor-stroma separation

## Abstract

Background: This study aims to explore the tumor-stroma separation or the cleft characterizing basal cell carcinoma (BCC).

Methodology: In this retrospective cohort investigation, we enrolled 244 patients who received a confirmed diagnosis of BCC through histopathological examination in the period of 2019-2020 at the Pathology Laboratory of the "Sfântul Apostol Andrei" Emergency Clinical Hospital located in Galați, Romania. The identification of patients was accomplished by utilizing electronic health records, and relevant clinical, demographic, and histopathological data were retrieved from the physical database of the Pathology Laboratory. Key tumor characteristics were gathered, and an in-depth analysis of case slides was performed.

Results: The average tumor-stroma cleft’s width measurement was 48.136 µm, while its respective tumor island’s width was on average 952.587 µm. The cleft’s width and its respective tumor island’s width are dependent on the BCC subtype, just like the ratio between the tumor island’s measurement and its cleft’s width are, being larger in basosquamous BCC, micronodular BCC, infiltrative BCC, and morpheaform BCC.

Conclusion: The BCC tumor islands were found to have a minimal approximately equal measurement to the tumor-stroma separation cleft, but they were always larger than the latter. Large clefts and their respective tumor islands were found in specific tumor subtypes such as basosquamous BCC, micronodular BCC, infiltrative BCC, and morpheaform BCC, but in nodular BCC also.

## Introduction

The term *tumor-stroma separation* or *tumor-stroma cleft* or the *clefting artifact* in the context of basal cell carcinoma (BCC) refers to a characteristic feature that can be observed during histopathological examination. By understanding the presence, extent, and purpose of this feature, one can provide important information for diagnosing and categorizing BCCs [1,2].

This notable histological feature, the tumor-stroma separation or cleft can be observed when examining a cross-section of BCC tumor, being seen as a gap (an empty space or an area containing a bluish substance (hyaluronic acid)) or separation between the tumor islands and the surrounding connective tissue/stroma [3], a feature that can help pathologists and dermatologists in the positive diagnosis of BCC and differentiate it from other skin lesions. It is also observable in superficial BCC and was previously interpreted as a retraction artifact in routine histopathology laboratory procedures. However, in other BCC types, like morpheaform/sclerosing, with a more fibrotic stroma and aggressive behavior, retraction spaces are typically absent [1,3-5]. The cleft is just one piece of the puzzle when it comes to diagnosing and treating this common form of skin cancer.

Newly emerging in vivo assessment methods, such as reflective confocal microscopy (RCM) and optical coherence tomography (OCT), both relying on light backscattering can be used either independently, as distinct clinical evaluation tools, or in an integrated manner, enhancing their capabilities. These techniques have shed light on the once-called *artificial cleft*, revealing that it may not be an outcome of routine procedures. Instead, it appears to be a *structure* or characteristic of BCC that is present in the patient's skin before any excision or processing takes place [1,6,7].

## Materials and methods

Study design and ethical considerations

The study adopted a retrospective cohort approach and took place at the "Sfântul Apostol Andrei" Emergency Clinical Hospital in Galați, Romania. Informed consent was obtained from all participating patients, and each consent form was securely attached to the respective patient records. The hospital’s Ethics Committee Approval was granted with the number 23903/October 30, 2023.

Patient selection

A total of 244 patients who were diagnosed and histologically confirmed to have BCC between January 1, 2019, and December 31, 2020, were included. Patients were identified through electronic health records, and their clinical and demographic data, along with histopathological information, were extracted from the Pathology Laboratory's physical database.

Data collection

Demographic information, encompassing age, gender, and race, was collected for all patients. Clinical data included the BCC subtype, lesion location, and any previous BCC history. Histopathological reports and case slides were meticulously reviewed and measurements were taken.

Pathological assessment

The histopathological analysis was carried out, and BCC subtypes were categorized in accordance with the World Health Organization (WHO) criteria. Subtypes included nodular, superficial, infiltrative, morpheaform, and micronodular. Tumor size was measured as the maximum dimension in micrometers. All of the necessary measurements were done by using a Meiji trinocular microscope with an Excelis video-photo camera and an ocular micrometer, the found values being introduced in an Excel file.

Statistical analysis

Statistical analysis was conducted using SPSS 27.0 (IBM Corp., Armonk, NY). Descriptive statistics, such as mean, standard deviation, and frequency distributions, were utilized to summarize patient characteristics and clinical variables. Various statistical tests, including chi-square tests, t-tests, Fisher tests, Kolmogorov-Smirnov tests, analysis of variance (ANOVA) tests, Mann-Whitney tests, and Kruskal-Wallis tests, were employed for comparing variables as appropriate. A *P*-value < 0.05 was considered statistically significant.

In conclusion, this comprehensive section outlines the study's design, data collection methods, laboratory procedures, statistical analysis, and outcome assessment, all of which were employed in our investigation of BCC. These methodologies were chosen to provide a solid foundation for the analysis of BCC characteristics, with the overarching objective of advancing our understanding of this common skin malignancy and determining the significance of tumor-stroma separation (clefting) in BCC by measuring the width of the cleft spaces and assessing their reliability as a histopathological marker for BCC.

## Results

The specific results of the current study have found that tumor-stroma clefts present an average width measurement of 48.136 µm (± 54.7362 µm). This tumor nest-surrounding stroma separation phenomenon has a rather extensive variation regarding its exact width, ranging from 5.5 µm to even 511.0 µm, the data being highlighted in Table 1. The latter also reports the findings on the measurement done regarding the cleft’s corresponding/respective tumor nest’s width which revealed that the cleft’s tumor nest widths varied from 29.0 µm to even 8500.0 µm (with an average measurement of 952.587 ± 1174.6217 µm).

**Table 1:** Measurements of the tumor-stroma separation cleft’s width and its tumor nest’s width, respectively (in µm).

Measurements	Number of patients	Max	Min	Average	Standard deviation
Tumor-stroma clefting space	244	511.0	5.5	48.1	54.7
Cleft’s respective tumor nest width	244	8500.0	29.0	952.5	1174.6

When taking the measurements of the tumor-stroma clefting space, the maximum width was discovered to be of actual statistical reliability according to the BCC’s tumor subtype, with a p value of 0.000, as it is highlighted in Table 2. The largest tumor-stroma clefting spaces (as in the widest, with the highest recorded values) were found in cases of nodular BCC, with mean values of 54.691 µm (± 60.2684 µm). The smallest/thinnest clefts were registered in cases of fibroepitheliomatous BCC (or Pinkus tumor), as they measured 10.500 µm.

**Table 2:** Largest cleft’s width and BCC tumor subtype correlation. Statistical test: Kruskal-Wallis, *H* = 27.8, *P* = 0.0 BCC, basal cell carcinoma

Tumor subtype	Largest cleft’s width				
	Number of patients	Min	Max	Mean	Standard deviation
Superficial BCC	3	14.7	32.6	21.4	9.7
Fibroepitheliomatous BCC	1	10.5	10.5	10.5	.
Superficial multicentric BCC	12	8.1	76.5	29.0	19.8
Nodular BCC	181	5.5	511.0	54.6	60.2
Basosquamous BCC	6	6.2	45.1	25.4	12.8
Micronodular BCC	15	7.4	171.9	39.9	38.8
Infiltrative BCC	22	8.1	106.2	26.2	26.5
Morpheaform BCC	4	17.2	32.5	23.6	6.3
Total	244	5.5	511.0	48.1	54.7

The current study has yielded interesting results regarding the dimensions of the widest cleft's respective tumor nests, which depend on the BCC tumor subtype in a statistically significant manner (*P* = 0.000), as revealed in Table 3. The largest/widest tumor nests corresponding to the largest/widest clefts were found in nodular BCC (as opposed to every other BCC subtype), with mean values of 1154.809 µm (with a standard deviation of 1280.9217 µm). The smallest tumor nests having the largest clefts were found in fibroepitheliomatous BCC (113.600 µm), closely followed by superficial BCC (203.033 ± 107.8040 µm) and superficial multicentric BCC (214.067 ± 163.0217 µm).

**Table 3:** Largest cleft’s respective tumor nest measurements and BCC tumor subtype correspondence. Statistical test: Kruskal-Wallis, *H* = 55.6, *P* = 0.0. BCC, basal cell carcinoma

Tumor subtype	Largest cleft’s respective tumor nest measurements				
	Number of patients	Min	Max	Mean	Standard deviation
Superficial BCC	3	130.4	326.9	203.0	107.8
Fibroepitheliomatous BCC	1	113.6	113.6	113.6	.
Superficial multicentric BCC	12	79.7	678.4	214.0	163.0
Nodular BCC	181	73.4	8500.0	1154.8	1280.9
Basosquamous BCC	6	141.8	1569.5	551.8	552.7
Micronodular BCC	15	43.1	2459.0	486.8	590.0
Infiltrative BCC	22	29.0	1866.3	346.6	383.2
Morpheaform BCC	4	214.1	1125.1	470.0	437.7
Total	244	29.0	8500.0	952.5	1174.6

Regarding the ratio between the cleft’s respective tumor nest’s and the cleft’s measurements (maximum width of the cleft), the current study reported values ranging from 1.04 to 317.77, having average values of 28.4022 (± 39.65101) (Table 4). Other significant data includes the median value of this ratio which was 15.2705, the inferior quartile having a value of 8.9922 (the range found in between 0.00 and this specific value covers 25% from all of the calculated values), while the superior one was registered at 30.9499 (the range found in between 0.00 and this specific value covering 75% from all calculated values).

The aforementioned ratio calculated between the largest cleft’s respective tumor nest and its largest cleft’s widths registered values that varied significantly among the tumor subtypes (p = 0.022). The highest ratios were found in nodular BCC 32.6966 (± 44.37777) while the lowest ones were registered in superficial multicentric BCC with 9.8158 (± 6.79144).

**Table 4:** The ratio between the cleft’s respective tumor nest’s and the largest cleft’s widths, according to the tumor subtype. Statistical test: Kruskal-Wallis, *H* = 16.3, *P* = 0.0. BCC, basal cell carcinoma

Tumor subtype	Number of patients	Min	Max	Mean	Standard deviation
Superficial BCC	3	4.0	22.2	11.7	9.4
Fibroepitheliomatous BCC	1	10.8	10.8	10.8	.
Superficial multicentric BCC	12	3.0	21.6	9.8	6.7
Nodular BCC	181	1.0	317.7	32.6	44.3
Basosquamous BCC	6	7.8	40.9	21.3	13.9
Micronodular BCC	15	1.4	65.2	15.4	16.2
Infiltrative BCC	22	2.1	67.3	18.4	18.4
Morpheaform BCC	4	8.8	50.0	20.6	19.6
Total	244	1.0	317.7	28.4	39.6

Regarding the cleft’s width of the largest BCC tumor nest, the current study has found that its average value was around 36.401 ± 48.8326 µm and that its variation range was in between 0.0 and 511.0 µm.

Of statistical relevance was the cleft’s width of the largest tumor nest by tumor subtype (*P* = 0.001), as revealed in Table 5. The highest values were found again in cases of nodular BCC (41.861 ± 53.8424 µm); the second subtype in which the largest clefts of the largest tumor nests were found was micronodular BCC (30.400 ± 41.4936 µm), while the smallest ones were identified also in the case of fibroepitheliomatous BCC (6.700 µm).

**Table 5:** The cleft’s width of the largest tumor nest by tumor subtype. Statistical test: Kruskal-Wallis, *H* = 24.5, *P* = 0.0. BCC, basal cell carcinoma

Tumor subtype	Number of patients	Min	Max	Mean	Standard deviation
Superficial BCC	3	10.1	14.7	12.1	2.3
Fibroepitheliomatous BCC	1	6.7	6.7	6.7	.
Superficial multicentric BCC	12	0.0	32.1	16.2	11.9
Nodular BCC	181	0.0	511.0	41.8	53.8
Basosquamous BCC	6	0.0	45.1	15.7	19.2
Micronodular BCC	15	0.0	171.9	30.4	41.4
Infiltrative BCC	22	7.1	92.5	19.7	17.8
Morpheaform BCC	4	11.5	32.5	20.7	9.0
Total	244			0.0	48.8

## Discussion

The tumor-stroma separation space, the cleft, or the clefting phenomenon is a characteristic tumor trait specific to BCC, serving as a diagnostic indicator when detected [8,9]. The precise mechanisms underlying the formation of these peritumoral clefts remain uncertain, with various theories proposed. While it was previously speculated that these retractions were the result of processing artifacts caused by fixation and dehydration [8,10], subsequent studies by multiple authors have refuted this notion and established the involvement of the tumor microenvironment. Ghita et al. utilized reflectance confocal microscopy to demonstrate the existence of in vivo peritumoral clefts. Ghita et al. noted the presence of dark spaces surrounding tumor islets [11-14]. These observations were further supported by Ulrich et al., who suggested the presence of mucin deposits within the peritumoral clefts [1,14]. Another study indicated that these spaces originate from the degradation of the extracellular matrix during tumor growth [15].

An alternative theory proposes that peritumoral retraction is a consequence of epithelial membrane disintegration. Several studies have shown a lack of laminin-5 in the area surrounding tumor nests, implying an improper structure or the absence of the hemidesmosome-anchoring filament complex in BCC, which results in the cleavage of the basal membrane [16,17]. The breakdown of the basal membrane was also demonstrated by Rios-Martin et al. through staining for Ep-CAM and cytokeratins [8]. However, not all researchers concur with this finding, as some argue that laminin does not significantly contribute to cleft formation [18].

More recent investigations have examined the impact of matrix metalloproteinases (MMPs), including MMP-2 and MMP-9, and have suggested that extracellular matrix remodeling is a significant factor, along with reduced expression of adhesion molecules [19]. Additionally, other metalloproteinases like stromelysin-3 have been implicated in tumor invasion through stromal matrix degradation [20]. Some researchers have observed increased MMP-2 expression in the stroma of high-risk BCC subtypes compared to low-risk subtypes, indicating a potential role in tumor invasiveness [21]. Nevertheless, when studying the presence of MMP-2 and MMP-9 in the peritumoral space, no statistically significant correlations were observed between these enzymes and the formation of peritumoral clefts [19].

The work at hand was carried out during the COVID-19 pandemic which has influenced patient care by lowering their hospital admittance or general medical care addressing. It has focused on studying BCC (a skin malignancy that is dependent on UV exposure but also exposure to other substances such as glucocorticoids, other photosensitizing drugs, arsenic, or ionizing radiation) [22] and the tumor-stroma separation space that can be found in such tumors and has revealed some interesting facts in regards to the cleft.

The current research has yielded significant results regarding the cleft found in BCC cases. The clefts investigated registered an average value of 48,136 µm, with a range of variation between 5.5 and 511 µm. The tumor nests corresponding to the measured clefts were found to have an average value of 952.587 µm, ranging from 29 to even 8500 µm. As such, the BCC nests were found to always have an approximately equal dimension to the cleft, but were always larger than the cleft itself, being 1.04 times to even 317.77 times larger.

Could it be possible that if the tumor islands are indeed larger than their respective cleft, the development of the latter is the simple consequence of a mechanical factor? The proliferation of the tumor islands is a continuous process but one that is also accompanied by cell apoptosis. The peripheral tumor island cells are arranged in a palisade (peripheral palisading cells) and are linked to the basal lamina with the help of hemidesmosomes. Taking into consideration the last two statements, due to the programmed cell death phenomenon certain strains develop, pulling forces that have an effect on the hemidesmosomes and act from inside the tumor island towards its periphery, the peripheral palisading basal cells giving in to the consequent mechanical stress. The aforementioned anomalies of the basal lamina (and surrounding stroma) might also add to the affected or reduced stress resistance, giving in to the development of the tumor-stroma separation which carries away the tumor island from the surrounding tissue. The apoptotic process is a programmed cell-death process innate to the cell, but it can also be induced by certain topical treatments like imiquimod [23,24], giving rise to the question of whether the cleft might potentially be modified with the help of local medication. It is well-established that tumor cells in BCC engage with the cellular and noncellular elements of the tumor microenvironment, fostering the advancement of tumors. The cellular components contribute to an immune-suppressed setting by dampening the activity of CD4+ and CD8+ T cells, while also encouraging the release of pro-oncogenic Th2 cytokines. This comprehension of the intricate interactions within the tumor microenvironment has driven the development of immunotherapeutic treatments, such as vismodegib for BCC [25]. The arrangement of biological cells responds not only to the biochemical composition of their surroundings but also to their mechanical characteristics. The interplay of the external medium referred to as the extracellular matrix, and its connection with transmembrane integrin proteins responsible for adhesion enables cells to explore the elasticity of their surroundings [26]. Research has found that a local cancer treatment might stem from an external mechanical stress applied to malignant cells (a theory tested on breast cancer cells), a factor that induced cell death [27]. Also, the cells within the tumor microenvironment encounter both external and internal pressures [28]; these mechanical stimuli from their surroundings are actively sensed by cells. In reaction to these mechanical stresses, they engage in mechanosensing and mechanotransduction processes, which are pivotal in controlling cell adhesion, shape, movement, growth, specialization, and migration. Cellular adhesions are influenced by cytoskeletal forces or external stresses, and they dynamically adjust to the mechanical traits of the surrounding extracellular matrix [29]. Besides the aforementioned mechanical stresses from the microenvironment, the mechanical characteristics of the extracellular matrix are also involved in cell processes, encompassing factors like its stiffness, structure, and adaptability [28,30]. The interaction between cells and the matrix exerts significant influence over various cellular functions. Additional research is imperative to gain insights into the key mechanical elements within the tumor microenvironment and the molecular mechanotransduction pathways. This will contribute to a deeper comprehension of the deformation, expansion, extrusion, and stiffening processes associated with solid tumor pathology, ultimately enhancing our understanding of cancer growth and metastasis [28,30]. The aforementioned processes might also be key elements in cleft development in BCC.

A rather striking finding which proved to be statistically reliable was the fact that the cleft’s width is a tumor trait that varies according to the tumor subtype. Thus, the largest clefts were found in nodular BCC. Other tumor subtypes that demonstrated large or wide clefts were basosquamous BCC, micronodular BCC, infiltrative BCC, and, surprisingly, superficial multinodular BCC. The largest cleft’s respective tumor island’s width was found to also vary according to the tumor subtype, the largest tumor islands being found in also in nodular BCC. Other BCC subtypes that proved to have large tumor islands with large clefts were basosquamous BCC, micronodular BCC, infiltrative BCC, and morpheaform BCC (all considered to be high risk).

The ratios between the widest cleft’s respective tumor islands and their cleft were found to be the highest in nodular BCC cases. Other tumor subtypes proved to have high ratios, such as basosquamous BCC, micronodular BCC, infiltrative BCC, and morpheaform BCC. This specific observation can also be drawn from the abovementioned discoveries regarding the measurements of the tumor islands and their clefts. This ratio registered also reduced values, this finding characterizing superficial multicentric BCC, a subtype that can present wide clefts but is associated with overall smaller tumor islands (as compared with other tumor subtypes).

It's important to acknowledge the limitations of this study, including its retrospective nature and reliance on historical medical records. Moreover, the generalizability of our findings might be influenced by the specific patient population at the "Sfântul Apostol Andrei" Emergency Clinical Hospital in Galați, Romania.

## Conclusions

The clefting phenomenon is a specific BCC trait. The BCC tumor islands were found to have a minimal approximately equal measurement to the tumor-stroma separation cleft, but they were always larger than the latter. Large clefts and their respective tumor islands were found in specific tumor subtypes such as basosquamous BCC, micronodular BCC, infiltrative BCC, and morpheaform BCC, but in nodular BCC also.
